# First Molecular and Physiological Characterization of Free-Living Amoebae from Environmental Water Sources in Honduras

**DOI:** 10.3390/tropicalmed11100271

**Published:** 2026-09-28

**Authors:** José Lisandro Mendoza-Munguía, Allisom Denise Lamothe Granados, Nahomy Alejandra Ruiz Dormes, Anderson Sered Bonilla Varela, Bryan Ortiz, Wilfredo Sosa-Ochoa, Soany Avilez, Karla Romero, Estefania Cuellar, Martín Cabello Vilchez

**Affiliations:** 1 Institute of Microbiology Research, Faculty of Sciences, National Autonomous University of Honduras, Tegucigalpa 11101, Honduraswilfredo.sosa@unah.edu.hn (W.S.-O.);; 2 School of Microbiology, Faculty of Sciences, National Autonomous University of Honduras, Tegucigalpa 11101, Honduras; allisom.lamothe@unah.edu.hn (A.D.L.G.);; 3 National Surveillance Laboratory, Ministry of Health, Tegucigalpa 11101, Honduras; 4 Laboratory of Pathogenic Protozoa and Endosymbionts, Alexander von Humboldt Institute of Tropical Medicine, Universidad Peruana Cayetano Heredia, Lima 15102, Peru; imtavh.protozoarios.amebas@oficinas-upch.pe

**Keywords:** free-living amoebae, *Acanthamoeba* spp., *Vermamoeba vermiformis*, Honduras, environmental surveillance

## Abstract

Free-living amoebae (FLA) are ubiquitous protozoa with pathogenic potential that also serve as environmental reservoirs for bacteria of public health importance. Owing to the lack of information on their occurrence in Honduras, this study aimed to isolate and characterize FLA from environmental water sources using morphological, molecular, and physiological approaches. A total of 87 water samples collected from domestic tap water, rivers, and outdoor washbasins were analyzed by non-nutrient agar culture, polymerase chain reaction, and 18S rRNA gene sequencing. Overall, 43 samples (49.4%) were positive for FLA, with the highest detection rate observed in domestic tap water (38/69; 55.1%). Molecular characterization of these 43 positive samples yielded 45 isolates in total, as two samples (codes 18.4 and 34.4) each produced two co-occurring but independently culturable isolates belonging to different genera. Of these 45 isolates, *Acanthamoeba* spp. accounted for 26 isolates (57.8%), predominantly belonging to genotype T4; *Vermamoeba vermiformis* accounted for 9 isolates (20.0%); and mixed cultures containing both genera accounted for 10 isolates (22.2%). A subset of *Acanthamoeba* isolates exhibited thermotolerance at 42 °C (7/26; 26.9%), with three isolates remaining viable at 45 °C, while most tolerated 0.5 M mannitol, and 57.7% retained viability at 1 M, characteristics commonly associated with pathogenic potential. To the best of our knowledge, this study constitutes the first report of free-living amoebae in environmental water sources in Honduras and provides an important baseline to strengthen water quality surveillance to mitigate potential public health risks.

## 1. Introduction

Free-living amoebae (FLA) are unicellular protists widely distributed in both natural and human-made environments [1]. They have been identified in a broad range of ecological niches, including soil, freshwater, brackish water, thermal waters, drinking water distribution systems, swimming pools, domestic water storage containers, and contact lens cases. Their occurrence across such diverse habitats reflects their remarkable ecological adaptability and frequent proximity to humans [2,3]. The ability of certain FLA to form highly resistant cysts enhances their survival under adverse conditions, including desiccation, environmental stress, and some disinfection procedures. This biological trait facilitates their persistence in aquatic systems of public health importance [4,5]. Moreover, FLA can grow and disperse in the environment independently of a host; consequently, the infections they cause are generally not transmitted from person to person [2]. Among the genera most frequently detected in environmental samples are *Acanthamoeba*, *Naegleria*, and *Vermamoeba*, whereas other genera, including *Vannella*, *Platyamoeba*, *Balamuthia*, and *Sappinia*, have been reported less frequently [6,7].

Within this context, *Acanthamoeba* spp. represents one of the most clinically, taxonomically, and epidemiologically significant genera of free-living amoebae [8]. Its molecular classification is primarily based on sequence analysis of the small subunit ribosomal RNA gene (18S rRNA), through which at least 23 genotypes (T1–T23) have been identified [9], including the recently described T23 genotype [10,11]. A further genotype, T24, has been very recently proposed based on whole-genome sequencing of a divergent Mexican isolate, although its formal validation by the broader scientific community is still pending [12]. Among these, the T4 genotype, belonging to the *Acanthamoeba castellanii* complex, is the most frequently detected in both environmental and clinical isolates [9,13,14]. This predominance is of epidemiological importance, as it suggests a close link between environmental reservoirs and the risk of human infection [8,9,15]. From a clinical perspective, *Acanthamoeba* has been implicated in *Acanthamoeba* keratitis, granulomatous amoebic encephalitis (GAE), and cutaneous infections [2,15]. Invasive infections occur predominantly in immunocompromised individuals, whereas keratitis can also affect immunocompetent patients, particularly contact lens wearers [9,16]. Despite their widespread environmental distribution, infections caused by these amoebae often present with nonspecific clinical manifestations, which may delay their recognition and timely diagnosis [15,17].

Beyond their direct pathogenic potential, FLA are of considerable microbiological and public health importance because of their interactions with other microorganisms [7]. As heterotrophic predators, they phagocytose bacteria, fungi, algae, and other microorganisms present in the environment [2,5,18]. However, some microorganisms can resist intracellular digestion, survive, and even replicate within amoebae [18]. This phenomenon has led to the concept of amoeba-resistant microorganisms (ARM), which includes several bacterial pathogens of public health concern, such as *Legionella* spp., *Pseudomonas aeruginosa*, and *Vibrio cholerae* [2,5,18]. Consequently, FLA can serve as intracellular reservoirs and environmental shelters for a wide range of pathogenic microorganisms, promoting their persistence and dissemination in the environment while protecting them from biocides and other adverse conditions [16,18]. The presence of potentially pathogenic FLA capable of harboring amoeba-resistant microorganisms is particularly relevant in tropical regions, where climatic conditions favor their persistence and proliferation in water sources used by humans. Honduras, a country characterized by a predominantly warm and humid climate and frequent human interaction with domestic, recreational, and natural aquatic environments, provides favorable conditions for continuous exposure to these protists. Nevertheless, information regarding the occurrence, distribution, and diversity of FLA in the country remains scarce. Studies describing the isolation and molecular characterization of FLA from environmental water sources are still lacking.

Therefore, the present study aimed to isolate and characterize FLA from diverse environmental water sources in Honduras using morphological, molecular, and physiological approaches, with particular emphasis on the identification of species of potential public health relevance. To the best of our knowledge, this study represents the first comprehensive environmental characterization of free-living amoebae in Honduras.

## 2. Materials and Methods

### 2.1. Sampling Sites

A total of 87 water samples were collected as part of an exploratory cross-sectional study based on convenience sampling from four departments of Honduras: Comayagua (*n* = 1), El Paraíso (*n* = 30), Francisco Morazán (*n* = 46), and Valle (*n* = 10). Samples were obtained from three types of water sources: rivers (*n* = 12), tap water (*n* = 69), and outdoor washbasins with stored water (*n* = 6). Most samples were collected from Francisco Morazán because of its greater geographic accessibility and the availability of sampling sites during the study period. The remaining departments were included to broaden the geographical coverage of the survey and to assess the occurrence of free-living amoebae across different regions of Honduras. These areas encompass diverse environmental and climatic conditions. Francisco Morazán and upland areas of El Paraíso are characterized by tropical highland climates and pine-oak forest ecosystems (elevations between ~700 and 1200 m a.s.l.), with mean annual temperatures of 20–23 °C. Comayagua represents an inter-montane valley (~550–650 m a.s.l.) with a dry tropical climate and mean temperatures of 24–26 °C. In contrast, Valle (<200 m a.s.l.) constitutes one of the warmest regions in the country, characterized by tropical dry forest ecosystems, mean annual temperatures of 28–30 °C (frequently exceeding 34–36 °C in daily maximums), and marked seasonal rainfall variation.

### 2.2. Sample Collection and FLA Isolation

Water samples were collected between May and August 2024 using sterile Whirl-Pak^®^ sampling bags (Nasco, Fort Atkinson, WI, USA). At each sampling site, 100 mL of water was collected. River and water storage samples were obtained by directly immersing the sterile bag into the water body, whereas tap water samples were collected directly into the sterile container until the required volume was reached. All samples were transported to the laboratory under refrigerated conditions (4 °C) and processed within 24–48 h of collection.

FLA isolation was performed following the protocols described by Lares-Villa and Hernández-Peña [19], with minor modifications. Briefly, 15 mL of each water sample was centrifuged at approximately 1100× *g* (4000 rpm) for 10 min. After discarding the supernatant, 100 μL of the resulting sediment was inoculated onto non-nutrient agar (NNA) plates overlaid with heat-inactivated *Escherichia coli* ATCC 25922 (80 °C for 30 min) as a food source. Bacterial inactivation was confirmed by plating aliquots of the heat-treated suspension on blood agar and MacConkey agar and verifying the absence of bacterial growth after incubation. Plates were labeled with an internal code corresponding to the sampling site and culture date and incubated at room temperature (25–28 °C). Cultures were examined after 24–48 h using light microscopy at 10× and 40× magnification for the presence of trophozoites and cysts, according to the morphological criteria described by Pussard and Pons [20].

Cultures initially identified as mixed were subjected to repeated subculturing until monocultures were obtained. Physiological assays and molecular analyses were performed exclusively on purified monogenic cultures (i.e., cultures purified to a single amoebal taxon by repeated subculturing; true axenization, defined as the absence of all associated bacteria and other microorganisms, was not experimentally confirmed). Mixed cultures that could not be successfully separated were excluded from physiological characterization. All cultures were monitored periodically for 15–30 days to confirm the presence or absence of amoebal growth.

### 2.3. DNA Extraction

Genomic DNA was extracted from all positive cultures. Briefly, each culture plate was washed with 0.85% sterile saline solution, and 2 mL of the resulting suspension was transferred to a microcentrifuge tube and centrifuged at 12,100× *g* (13,000 rpm) for 2 min to obtain a cell pellet. Genomic DNA was extracted from the pellet using the Wizard^®^ Genomic DNA Purification Kit (Promega, Madison, WI, USA) according to the manufacturer’s instructions. DNA concentration was determined using a UV5Nano spectrophotometer (Mettler Toledo^®^, Columbus, OH, USA).

### 2.4. PCR Amplification and Molecular Characterization of FLA Isolates

The initial molecular screening of free-living amoebae (FLA) was performed using the universal Free-Living Amoeba (FLA-F/FLA-R) primer pair targeting the 18S rRNA gene. These primers amplify fragments ranging from approximately 750 to 1100 bp, depending on the genus or species detected.

Cultures exhibiting morphological characteristics consistent with *Vermamoeba* spp. and producing amplicons of approximately 750–800 bp with the FLA primers were subjected directly to PCR product purification and Sanger sequencing for species confirmation as *Vermamoeba vermiformis*. In contrast, isolates with morphological features suggestive of *Acanthamoeba* spp. and amplicons ranging from 800 to 1100 bp were further analyzed by a second PCR using the genus-specific JDP1/JDP2 primer pair. This assay amplifies a fragment of approximately 450 bp corresponding to the hypervariable Diagnostic Fragment 3 (DF3) region of the 18S rRNA gene. The resulting amplicons were subsequently purified and sequenced to confirm the genus and assign the corresponding genotypes (T1–T24). The overall molecular workflow and decision-making strategy are summarized in Figure 1.

The primers used in this study, together with their nucleotide sequences, annealing temperatures (*T_m_*), and corresponding references, are presented in Table 1.

Conventional PCR assays were performed in a final reaction volume of 50 μL containing 25 μL of PCR Master Mix (Promega Corp., Madison, WI, USA), 1 μL each of the forward and reverse primers (10 μM), and template DNA. Amplifications were carried out under the following cycling conditions: an initial denaturation at 95 °C for 5 min, followed by 35 cycles of denaturation at 95 °C for 30 s, primer annealing at the temperature specific for each primer set (Table 1) for 30 s, and extension at 72 °C for 1 min, with a final extension at 72 °C for 7 min. PCR products were separated by electrophoresis on 2% agarose gels stained with ethidium bromide. A 100-bp DNA ladder was used as the molecular size marker. DNA bands were visualized using a Gel Doc™ EZ Imager UV transilluminator (Bio-Rad, Hercules, CA, USA). For each PCR run, DNA extracted from a sample that had previously produced a strong, unambiguous amplicon and whose identity had been independently confirmed by Sanger sequencing was used as an internal positive control (samples 3.2 and 37.4; see Table 2 for their taxonomic identification).

### 2.5. PCR Product Sequencing and Sequence Analysis

To confirm the molecular identity of the different PCR amplification patterns, a total of 25 PCR amplicons derived from 21 representative amoebal cultures were selected for Sanger sequencing at Psomagen Inc. (Rockville, MD, USA), using the FLA primers (*n* = 10) and JDP primers (*n* = 15). Four isolates (codes 3.2, 6.2, 18.4, and 1.3) were sequenced using both primer sets, yielding two independent GenBank accessions each. The resulting sequences were assembled and edited using Geneious^®^ (v2023.2.1), and their taxonomic identities were confirmed by BLAST (https://blast.ncbi.nlm.nih.gov/Blast.cgi, accessed between 1 October 2025 and 30 June 2026) searches against the NCBI nucleotide database. The validated sequences were subsequently deposited in GenBank (https://www.ncbi.nlm.nih.gov/genbank/, accessed between 1 October 2025 and 30 June 2026).

### 2.6. Phylogenetic Analysis

Consensus sequences generated in this study were aligned with reference sequences retrieved from the GenBank database using MAFFT (v7.505) [23] and Geneious Prime^®^ (version 2025.2.2; Biomatters Ltd., Auckland, New Zealand). Best-fit substitution models were selected using ModelFinder (implemented in IQ-TREE2 v2.0.7) [24,25] and, independently, using the built-in model set of RAxML (v8.2.11) [26], which is restricted to GTR-based models. Maximum Likelihood (ML) phylogenetic trees were reconstructed using RAxML under the GTR+GAMMA model, with tree search and rapid bootstrapping performed simultaneously (1000 replicates; “search for best-scoring ML tree” algorithm). As an independent cross-validation, ML trees were also inferred using IQ-TREE2 under the ModelFinder-selected model (Bayesian Information Criterion). Tree rooting was performed using taxon-specific outgroups selected within Amoebozoa or, when appropriate, within the same genus, to avoid the long-branch attraction associated with highly divergent outgroups. For the *Acanthamoeba* analysis based on the partial 18S rRNA region amplified with the FLA-F/FLA-R primers, *Balamuthia mandrillaris* isolate V039 (GenBank accession number AF477019) was used as the outgroup, as this taxon belongs to a closely related amoebozoan lineage [27]. For the *Acanthamoeba* analysis based on the hypervariable DF3 region amplified with the JDP1/JDP2 primers, an external outgroup was avoided because this genus-specific, highly variable fragment could not be reliably aligned across distantly related taxa; instead, the tree was rooted using *Acanthamoeba lenticulata* strain PD2S (genotype T5; GenBank accession number U94741), which was independently confirmed as the most basal *Acanthamoeba* genotype in the full-length 18S rRNA phylogeny. For the *Vermamoeba vermiformis* analysis, *Balamuthia mandrillaris* isolate V039 (GenBank accession number AF477019) was used as the outgroup. Node support was assessed using ultrafast bootstrap approximation/rapid bootstrapping with 1000 replicates; only values ≥ 50% were retained in the consensus trees.

### 2.7. Physiological Assays

#### 2.7.1. Thermotolerance Assay

Following confirmation of isolate identity by morphological examination and PCR, thermotolerance was assessed. Monocultures were inoculated onto non-nutrient agar (NNA) plates overlaid with a heat-inactivated *Escherichia coli* lawn and incubated at 37 °C, 42 °C, and 45 °C. Cultures were examined microscopically after 48, 72, and 96 h, with the presence of motile trophozoites used as the criterion for viability. To confirm cell survival or death, cultures were subsequently subcultured onto fresh NNA plates and incubated at room temperature to evaluate their ability to excyst, proliferate, and undergo normal re-encystment. Each assay was performed in duplicate.

#### 2.7.2. Osmotolerance Assay

Osmotolerance was evaluated by incubating monocultures on non-nutrient agar (NNA) supplemented with mannitol at final concentrations of 0.5 M and 1 M and overlaid with a heat-inactivated *Escherichia coli* lawn at 30 °C. Cultures were examined microscopically after 48, 72, and 96 h, using the presence of motile trophozoites as the criterion for viability. To confirm amoebal survival, cultures were subsequently subcultured onto mannitol-free NNA plates and incubated under ambient conditions to verify population recovery and encystment dynamics. Each assay was performed in duplicate.

## 3. Results

### 3.1. Sampling Sites and Isolation of Free-Living Amoebae

Of the 87 water samples analyzed, 43 (49.4%) were positive for free-living amoebae (FLA) growth on non-nutrient agar (NNA) supplemented with heat-inactivated *Escherichia coli*. The highest number of positive isolates was obtained from the department of Francisco Morazán (37/46; 80.4%), followed by El Paraíso (3/30; 10.0%), Valle (2/10; 20.0%), and Comayagua (1/1; 100%). According to water source type, most positive isolates were recovered from tap water samples (38/69; 55.1%), although FLA were also detected in outdoor washbasins supplied with stored water (3/6; 50.0%) and river water samples (2/12; 16.7%).

### 3.2. Morphological and Molecular Characterization of the Isolates

Viable isolates exhibiting morphological features consistent with free-living amoebic trophozoites and cysts were recovered from the positive cultures and serially subcultured to obtain purified monogenic cultures free of contaminating microorganisms. Preliminary microscopic examination revealed morphological characteristics consistent with the genera *Acanthamoeba* and *Vermamoeba*. *Acanthamoeba* spp. isolates exhibited active trophozoites bearing characteristic spine-like cytoplasmic projections (acanthopodia) and distinctive double-walled cysts with polygonal or stellate endocysts. In contrast, *Vermamoeba vermiformis* was characterized by elongated cylindrical trophozoites displaying directional movement (limax morphology) and spherical to ovoid cysts with a smooth, single-layered wall, frequently observed in dense aggregates. Representative micrographs illustrating these diagnostic morphological features are shown in Figure 2.

The molecular characterization based on amplification of a partial region of the 18S rRNA gene identified 26 *Acanthamoeba* spp. isolates (57.8%), nine *Vermamoeba vermiformis* isolates (20.0%), and ten mixed cultures (22.2%). Mixed cultures were defined by the simultaneous detection of genus-specific PCR amplicons for both *Acanthamoeba* spp. and *V. vermiformis* within the same culture, indicating the coexistence of both amoebae. Representative PCR amplification profiles obtained using the FLA and JDP1/JDP2 primer sets are shown in Figure 3.

From these PCR-positive isolates, 21 representative isolates were selected for confirmation by Sanger sequencing. Their taxonomic identities are summarized in Table 2.

### 3.3. Physiological Characterization of the Isolates

The thermotolerance and osmotolerance assays revealed differences in the physiological tolerance of the genera evaluated (Table 2). A total of 10 mixed cultures could not be assessed because purified monogenic cultures could not be obtained. Cell viability was defined by the presence of active, motile trophozoites and sustained recovery/re-encystment capacity upon subculturing. Among the *Acanthamoeba* spp. isolates (*n* = 26), 24 (92.3%) grew at 37 °C, 7 (26.9%) at 42 °C, and 3 (11.5%) at 45 °C. In the osmotolerance assay, 19 isolates (73.1%) tolerated 0.5 M mannitol, whereas 15 (57.7%) remained viable at 1 M. In contrast, *Vermamoeba vermiformis* (*n* = 9) exhibited lower physiological tolerance: 7 isolates (77.7%) grew at 37 °C, only 1 (11.1%) at 42 °C, and none at 45 °C. Likewise, 4 isolates (44.4%) tolerated 0.5 M mannitol, whereas none survived at 1 M. Notably, isolates that did not grow under any of the temperature or osmotic conditions evaluated (e.g., 7.4, 12.4, 34.4, 37.4, and 5.5) remained viable at room temperature (25–28 °C) throughout the study.

### 3.4. Phylogenetic Analysis

#### 3.4.1. Phylogenetic Analysis of Acanthamoeba spp. Based on the Partial 18S rRNA Gene Region (FLA-F/FLA-R Primers)

Phylogenetic analysis based on the FLA-F/FLA-R amplicons (Figure 4) assigned the local isolates to genotypes T4, T1, and the T2/T6 complex (see Supplementary File S1). Two well-supported subclades were identified within the T4 genotype: FLA 1.3/FLA 3.2 (99% bootstrap support) and FLA 6.2/FLA 1.2 (97% bootstrap support). In contrast, FLA 30.1 was positioned basal to genotype T1 (88% bootstrap support), and FLA 18.4 grouped within the T2/T6 complex (80% bootstrap support). The Honduran sequences were interspersed among global reference strains, clustering closely with clinical isolates associated with *Acanthamoeba* keratitis, pulmonary infections, and granulomatous amoebic encephalitis (GAE).

#### 3.4.2. Analysis of the Hypervariable DF3 Region of *Acanthamoeba* spp. (JDP1/JDP2 Primers)

Phylogenetic analysis of the DF3 region (Figure 5) confirmed the genotype assignments with variable resolution (see Supplementary File S2). JDP-amplified isolates corresponding to the T4 *A. castellanii* lineage (JDP 3.2, 6.3, 18.3, 15.1, 35.4, and 36.4) clustered together with low-to-moderate support (23–51% bootstrap support). JDP 21.4 (*A. palestinensis*) and JDP 13.4 (*A. hatchetti*) grouped with T3 reference sequences associated with human ocular infections (*Acanthamoeba* keratitis; 94% bootstrap support). Notably, isolates associated with the T2/T6 complex (JDP 34.4, 18.4, 19.4, 28.4, 6.4, and 1.3) formed a well-supported monophyletic clade (95% bootstrap support), providing a clearer resolution of this genotype complex, demonstrating the discriminatory power of the DF3 region for genotype identification in isolates recovered from domestic water sources.

#### 3.4.3. Identification and Phylogenetic Analysis of Vermamoeba Vermiformis

The phylogenetic tree of *Vermamoeba vermiformis* (Figure 6) placed the Honduran isolates within the broader global diversity of this species, forming two distinct, well-supported pairs rather than a single unique lineage: FLA 18.4/FLA 34.4 (93% bootstrap support) and FLA 39.4/FLA 30.4 (98% bootstrap support), each nested among international reference sequences (see Supplementary File S3). This pattern is consistent with the limited genetic divergence reported among globally distributed *V. vermiformis* strains at the 18S rRNA locus.

## 4. Discussion

To the best of our knowledge, this study represents the first report describing the isolation and molecular characterization of free-living amoebae from environmental water sources in Honduras. The overall positivity rate observed (49.4%; 43/87 samples) indicates widespread occurrence of these protozoa in the water sources examined. When compared with studies conducted on drinking water distribution systems and domestic water supplies in tropical and subtropical regions, the prevalence observed in Honduras is considerably higher. For example, studies from Southeast Asia have reported isolation frequencies ranging from 2.4% to 33.3% in tap water and household water storage tanks [28], whereas investigations of hospital water distribution systems and urban water supplies in the Middle East have documented prevalences ranging from 21.1% to more than 80% [29,30]. The high frequency of FLA detected in domestic water sources in Honduras not only exceeds that reported for most household water supply systems worldwide but is also comparable to the prevalence typically described in untreated environmental water reservoirs [30]. These findings suggest potential deficiencies in water treatment processes and/or environmental conditions that favor persistent colonization and long-term establishment of FLA within the water distribution network [3,31].

The most striking finding was the 55.1% positivity rate (38/69) observed exclusively in tap water samples. This prevalence exceeds those reported for urban water distribution systems in Southeast Asia, where isolation rates range from 2.4% to 33.3% depending on the country and water supply system evaluated [28]. Furthermore, it is higher than values reported in neighboring Central American countries, such as Nicaragua, where a 23% prevalence of free-living amoebae was found in tap water [32], and Costa Rica, where positivity rates around 30% have been documented in domestic and healthcare water supply systems [33,34]. In contrast, our findings closely resemble those reported by Lorenzo-Morales et al. in Tenerife, Canary Islands (Spain), who identified a 59.5% positivity rate for *Acanthamoeba* spp. in tap water [35].

This discrepancy may be explained by the convergence of several factors characteristic of the Honduran context, including an intermittent drinking water supply that necessitates household water storage in cisterns and outdoor washbasins, aging and potentially corroded water distribution infrastructure, and widespread biofilm formation during periods of water stagnation. These biofilms provide ideal ecological niches for FLA by offering a stable organic matrix, protection from residual disinfectants, and access to bacterial communities that serve as a food source [28]. Moreover, the consistently warm tropical climate of Honduras, where mean annual temperatures typically exceed 24 °C, provides favorable thermal conditions for the persistence and proliferation of thermotolerant genera such as *Acanthamoeba* spp. [36].

In terms of taxonomic composition, molecular characterization identified *Acanthamoeba* spp. as the predominant genus (26/45 isolates; 57.8%), followed by *Vermamoeba vermiformis* (9/45; 20.0%) and mixed cultures containing both genera (10/45; 22.2%). This distribution is consistent with multicenter studies reporting *Acanthamoeba* spp. as the most ubiquitous free-living amoeba genus in aquatic environments [37,38,39]. The frequent detection of both genera in positive cultures (22.2%) is ecologically relevant, as it suggests the coexistence of diverse amoebal populations within shared biofilm communities. Previous studies have demonstrated that *Acanthamoeba* and *V. vermiformis* exhibit overlapping ecological niches and can coexist within complex microbial consortia in water distribution systems [40]. Notably, in hospital water systems, *V. vermiformis* has even been reported more frequently than *Acanthamoeba* spp. [30,41], underscoring the importance of including this species in microbiological surveillance programs for water quality. The detection of *V. vermiformis* in 20.0% of the isolates (9/45) is of particular significance because this amoeba is recognized as a host for several clinically important bacterial pathogens, including *Legionella pneumophila*, *Pseudomonas aeruginosa*, and *Bacillus anthracis*, and has been reported to occur at higher densities than *Acanthamoeba castellanii* in drinking water and associated biofilms [18,30]. Its isolation from tap water in Honduras therefore reinforces the hypothesis that free-living amoebae should be regarded not only as potential opportunistic pathogens but also as environmental reservoirs for bacteria of public health importance [42].

Molecular characterization based on the hypervariable DF3 region (JDP1/JDP2 primers) revealed the predominance of genotype T4 among the *Acanthamoeba* isolates, with all sequences clustering within the *A. castellanii* complex. This finding is consistent with a previous systematic review of 427 environmental isolates, which reported genotype T4 as the most prevalent (approximately 48%), followed by T3 (13%), T5 (13%), and T2 (11%) [43]. Likewise, Fabros et al. identified T4 as the predominant genotype in thermal spring environments worldwide [44], while studies conducted in Iran, Egypt, Thailand, and Türkiye have consistently confirmed T4 as the dominant genotype in environmental water samples [37,45,46]. Similar findings have also been reported in Latin America, where studies from Peru [47] and Mexico [44] documented genotype T4 as the dominant genotype in both environmental and clinical isolates. Together, these observations reinforce the global ecological success of genotype T4 and its remarkable adaptability to diverse aquatic environments.

The identification of *A. castellanii* and *A. hatchetti* within this genotype/complex, together with a single isolate of *A. palestinensis*, is consistent with the well-established association between *A. castellanii* and genotype T4 in urban aquatic environments worldwide [10,48]. Notably, *A. hatchetti*, originally isolated from marine sediments in Baltimore Harbor, has demonstrated pathogenic potential in experimental murine models and has been implicated as an etiological agent of granulomatous amoebic encephalitis (GAE) in humans. Consequently, its detection in Honduran tap water extends the clinical relevance of the present findings, given the routine human exposure associated with domestic water use [49,50].

Among the remaining genotypes, the identification of an isolate (FLA 30.1) positioned basally to genotype T1 is of particular interest. Although T1 is infrequently recovered from environmental sources, it has been associated with central nervous system infections, including granulomatous amoebic encephalitis (GAE) [8]. Phylogenetic analysis showed that isolate FLA 30.1 clustered closely with reference sequences derived from clinical GAE cases, suggesting that genetically related lineages may also occur in domestic water environments in Honduras. Although this isolate was purified to a single amoebal taxon but not experimentally confirmed as axenic (i.e., free of associated bacteria), which precludes definitive conclusions regarding its pathogenic potential, its detection is noteworthy given previous reports indicating that T1 strains have been recovered predominantly from brain tissue, with only limited environmental documentation [8]. In contrast, sequences assigned to the T2/T6 lineages and *A. hatchetti* clustered predominantly with environmental reference strains from Europe and the Middle East, providing no evidence of geographically endemic clades in Honduras and further supporting the cosmopolitan distribution of the genus. Moreover, the clustering of the Honduran T4 isolates with international clinical strains associated with *Acanthamoeba* keratitis (e.g., U07414.1 and U07405.1) further supports the close phylogenetic relationship between environmental and clinical *Acanthamoeba* isolates, reinforcing the role of aquatic environments as important reservoirs of potentially pathogenic strains [8,48].

From a physiological perspective, the finding that 92.3% (24/26) of the purified monogenic *Acanthamoeba* isolates exhibited active growth at 37 °C is noteworthy, as this temperature corresponds to that of the human body and has been proposed as an in vitro indicator of pathogenic potential [36,38]. In contrast, only 26.9% (7/26) tolerated 42 °C, and just three isolates (11.5%; 15.1, 17.4 and 21.4) remained viable at 45 °C. This pattern agrees with previous studies demonstrating that while growth at 37 °C is common among environmental isolates, thermotolerance above 40 °C is considerably less frequent [46,51]. The identification of three highly thermotolerant isolates is particularly relevant because tolerance to elevated temperatures has been associated with increased virulence and enhanced adaptation to host tissues [36,38,46]. Notably, isolates (15.1, identified as *A. castellanii*; 17.4, identified as *Acanthamoeba* spp.; and 21.4, identified as *A*. *palestinensis*) were recovered from tap water intended for human consumption, highlighting the potential public health implications of chronic exposure to environmental strains that exhibit characteristics associated with pathogenicity [10,46].

From an osmotolerance perspective, 73.1% (19/26) of the *Acanthamoeba* isolates grew in the presence of 0.5 M mannitol, whereas 57.7% (15/26) remained viable at 1 M. These findings are consistent with those of Üstüntürk-Onan, who reported that both *Acanthamoeba* isolates recovered from well water in Istanbul tolerated 1 M mannitol but failed to grow at 42 °C and were therefore considered to exhibit low pathogenic potential [52]. The high level of osmotolerance observed in the present study may reflect adaptation to hyperosmotic microenvironments, a characteristic that has been associated with enhanced survival on the ocular surface and may facilitate the establishment of corneal infection [36]. Nevertheless, pathogenicity in *Acanthamoeba* is a multifactorial trait influenced not only by physiological characteristics but also by genotype and a wide range of molecular virulence determinants. Accordingly, combining physiological markers with molecular characterization and functional assays, including cytotoxicity testing and experimental infection models, will be essential for a more comprehensive assessment of the pathogenic potential of these isolates [11].

Compared with *Acanthamoeba*, *V. vermiformis* isolates exhibited lower physiological tolerance: 77.7% (7/9) of the isolates grew at 37 °C, only one isolate (11.1%; 20.4) tolerated 42 °C, and none remained viable at 45 °C. Osmotolerance was even more limited, with only 44.4% (4/9) of the isolates growing in the presence of 0.5 M mannitol and none surviving at 1 M. These observations agree with previous reports describing *V. vermiformis* as less tolerant to thermal and osmotic stress than *Acanthamoeba* spp. [18,40]. However, the observation that five isolates (7.4, 12.4, 34.4, 37.4, and 5.5) remained viable at room temperature (25–28 °C) despite failing to survive under the experimental conditions should be interpreted with caution, as their ability to encyst may confer resistance to environmental fluctuations without necessarily indicating metabolic activity under stressful conditions. Nevertheless, the public health relevance of *V. vermiformis* derives primarily from its established role as an environmental host for pathogenic bacteria and its documented involvement in polymicrobial keratitis together with *Acanthamoeba* spp., rather than from intrinsic thermotolerance [18,30,40]. Therefore, its detection in 20.0% of pure isolates and 22.2% of mixed cultures highlights its potential epidemiological importance in domestic water systems.

Another noteworthy finding was the absence of *Naegleria fowleri*, despite the tropical climate of Honduras, which provides environmental conditions generally considered favorable for this thermophilic amoeba [53]. This contrasts with reports from neighboring countries, including Mexico, where *N. fowleri* has been detected in swimming pools and other aquatic environments [19]. Several factors may explain this observation. First, competition during culture on non-nutrient agar may favor the rapid overgrowth of *Acanthamoeba* spp. and *V. vermiformis*, thereby reducing the likelihood of recovering *N. fowleri* [50,53]. Second, environmental detection of *N. fowleri* typically peaks during periods of extreme heat, when water temperatures exceed 30 °C, and under stagnant water conditions [53]. Although the sampling period in the present study (May–August) included warm months, the temporal sampling frequency and the number of environmental sampling sites may have limited the likelihood of detecting this species. Future studies could overcome these limitations by incorporating selective culture media with elevated incubation temperatures (42–45 °C) and antibiotic supplementation, together with culture-independent approaches such as quantitative PCR directly from filtered water samples. These complementary methods would likely provide a more accurate estimate of the environmental occurrence of *N. fowleri* in Honduras, particularly under future climate scenarios expected to expand its ecological niche.

From a public health perspective, the detection of genotype T4 *Acanthamoeba* strains—the principal cause of *Acanthamoeba* keratitis (AK) and the genotype most frequently associated with granulomatous amoebic encephalitis (GAE) [43,44,54,55]—in domestic tap water in Honduras establishes a plausible environmental source of human exposure. Previous studies conducted in the United Kingdom, Hong Kong, and the United States have demonstrated, through molecular typing, direct links between corneal isolates from patients with AK and *Acanthamoeba* strains recovered from the household water supplies of the same individuals [56,57,58]. Although clinical cases of AK or GAE have not yet been documented in Honduras, the presence of these clinically important genotypes in domestic water suggests a latent public health risk, particularly for contact lens wearers who use tap water to rinse or store their lenses. Accordingly, preventive measures, including the exclusive use of sterile saline or commercially approved contact lens solutions, routine replacement of lens storage cases, and avoidance of tap water exposure, should be emphasized as key public health recommendations in the Honduran setting [59].

Additionally, the high frequency of mixed cultures containing *Acanthamoeba* and *V. vermiformis* (22.2%) has broader implications for the microbiological quality of domestic water supplies. Complex amoebal communities not only increase the biological diversity of biofilms but also facilitate the persistence, replication, and horizontal transfer of intracellular bacteria [28,42]. In countries where infections caused by *Legionella* spp. and *Pseudomonas aeruginosa* represent significant clinical challenges, the presence of free-living amoebae capable of harboring these opportunistic pathogens in domestic water may increase the likelihood of human exposure. Collectively, these findings support the inclusion of FLA monitoring within drinking water surveillance programs, particularly in regions where environmental conditions favor their persistence [6,7]. Such surveillance could incorporate seasonal monitoring of water distribution systems, molecular characterization of isolates to identify clinically relevant genotypes, integration of environmental and hospital-based surveillance data, and public health education promoting safe household water storage and contact lens hygiene.

The findings of this study should be interpreted in light of several limitations. Sampling was concentrated primarily in the Department of Francisco Morazán (46/87 samples), introducing geographic bias and limiting extrapolation to the national level. Nevertheless, Francisco Morazán encompasses the capital city and the largest population center in Honduras, making these findings particularly relevant from an urban public health perspective. In addition, the limited number of river water (*n* = 12) and outdoor water storage samples (*n* = 6) precludes robust conclusions regarding the diversity and distribution of free-living amoebae in non-domestic aquatic environments. Genotype assignment was restricted to isolates that achieved a purified monogenic culture and yielded DNA of sufficient quality, resulting in the exclusion of numerous persistent mixed cultures. Additionally, genotype assignment for the JDP1/JDP2-amplified isolates relied exclusively on the partial, hypervariable DF3 region of the 18S rRNA gene. While this fragment offers adequate resolution for major, well-differentiated genotype groups such as T4, it may provide limited discriminatory power for closely related genotypes (e.g., within the T2/T6 complex) and for isolates with ambiguous or basal phylogenetic placement, such as FLA 30.1 (positioned basally to genotype T1). Genotype assignments based solely on the DF3 fragment should therefore be interpreted with appropriate caution, and confirmation using longer or near-complete 18S rRNA sequences is recommended for isolates of uncertain taxonomic placement. This limitation was directly observed in four isolates (codes 3.2, 6.2, 18.4, and 1.3) that were sequenced using both primer sets: species-level identification (e.g., A. castellanii) was consistently achieved with the near-complete 18S rRNA fragment (FLA-F/FLA-R), whereas the shorter DF3 fragment (JDP1/JDP2) resolved these same isolates only to the genus level (Acanthamoeba sp.), underscoring the reduced discriminatory power of the DF3 region for species-level identification despite its utility for genotype assignment. Future studies should therefore implement more efficient axenization protocols or employ direct metagenomic sequencing of environmental DNA to improve the characterization of mixed amoebal communities. Finally, neither cell culture cytopathogenicity assays nor in vivo infection models were performed to directly evaluate the virulence of the isolates. Consequently, the pathogenic potential discussed in the present study is inferred from indirect indicators, including thermotolerance, osmotolerance, species identification, and genotype.

Overall, this study provides the first comprehensive molecular, morphological, and physiological characterization of free-living amoebae in environmental water sources in Honduras. The predominance of genotype T4 *Acanthamoeba* in domestic tap water, together with the frequent detection of *V. vermiformis* and the identification of isolates exhibiting physiological characteristics associated with pathogenicity, provides the first evidence that domestic water systems in Honduras may serve as environmental reservoirs of clinically relevant free-living amoebae. These findings establish an important baseline for future nationwide surveillance and contribute to a more comprehensive understanding of the potential public health risks posed by these microorganisms.

## 5. Conclusions

This study presents the first molecular, morphological, and physiological characterization of free-living amoebae (FLA) in environmental water sources in Honduras. A high prevalence of FLA was detected, particularly in domestic tap water, with *Acanthamoeba* spp. identified as the predominant genus, followed by *Vermamoeba vermiformis* and mixed cultures containing both genera. Molecular analysis demonstrated the predominance of genotype T4, the genotype most frequently associated with *Acanthamoeba* keratitis and granulomatous amoebic encephalitis, while also identifying isolates affiliated with the T1 lineage and *A. palestinensis*. Furthermore, several *Acanthamoeba* isolates exhibited high thermotolerance and osmotolerance, physiological characteristics commonly associated with pathogenic potential. Collectively, these findings indicate that domestic water systems in Honduras constitute potential environmental reservoirs of clinically relevant free-living amoebae and underscore the importance of incorporating FLA into environmental water quality surveillance and public health monitoring strategies, particularly for populations at increased risk, such as contact lens users and immunocompromised individuals.

## Figures and Tables

**Figure 1:**
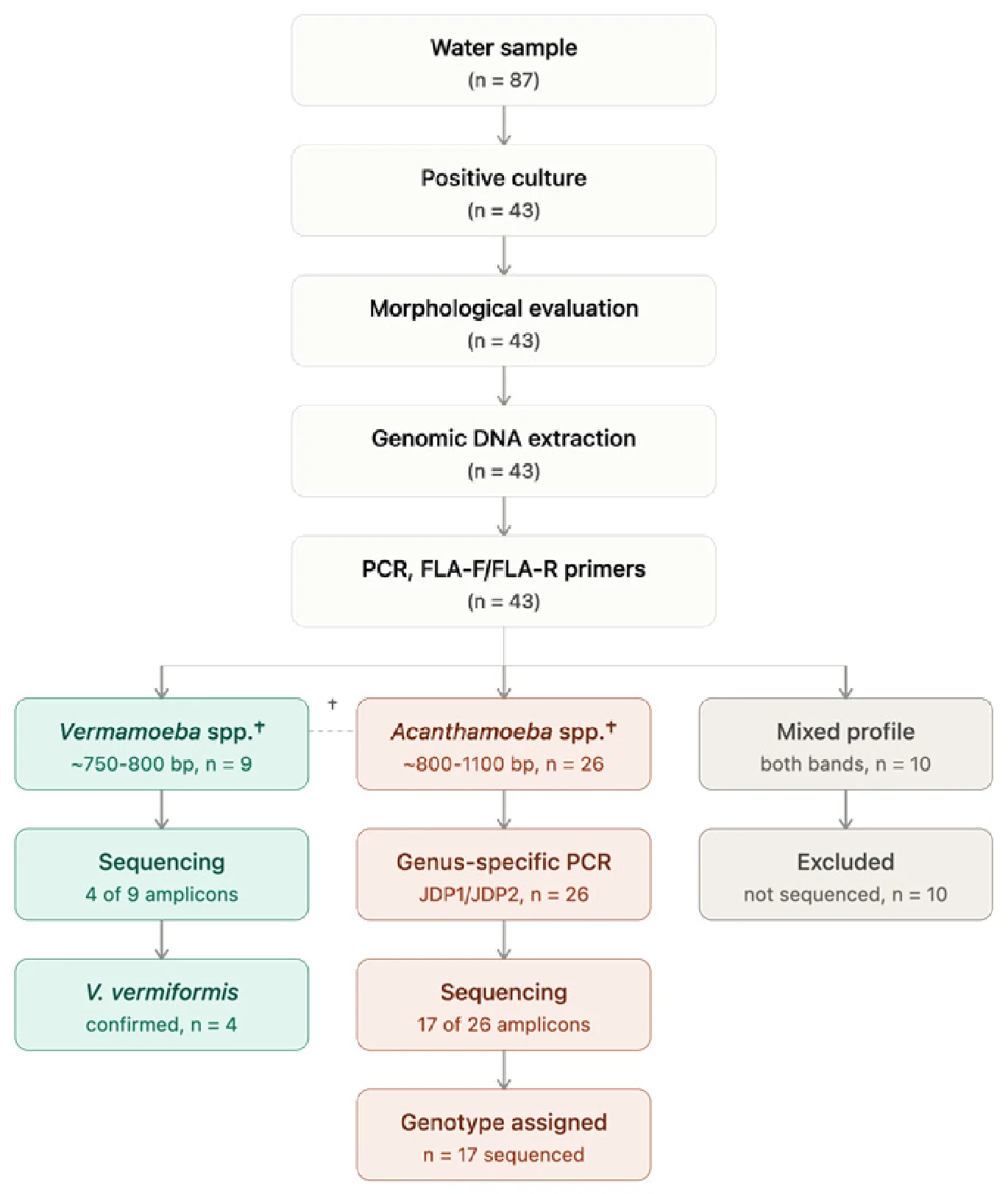
Quantitative molecular workflow and decision algorithm used for the identification of free-living amoebae from environmental water samples, detailing sample counts at each processing stage. The dagger symbol (†) indicates that two samples (codes 18.4 and 34.4) each yielded isolates of both genera and are counted in both branches (total *n* = 45 isolates from 43 positive samples). Four *Acanthamoeba* isolates (codes 3.2, 6.2, 18.4, and 1.3) were sequenced using both the FLA and JDP primer sets.

**Figure 2:**
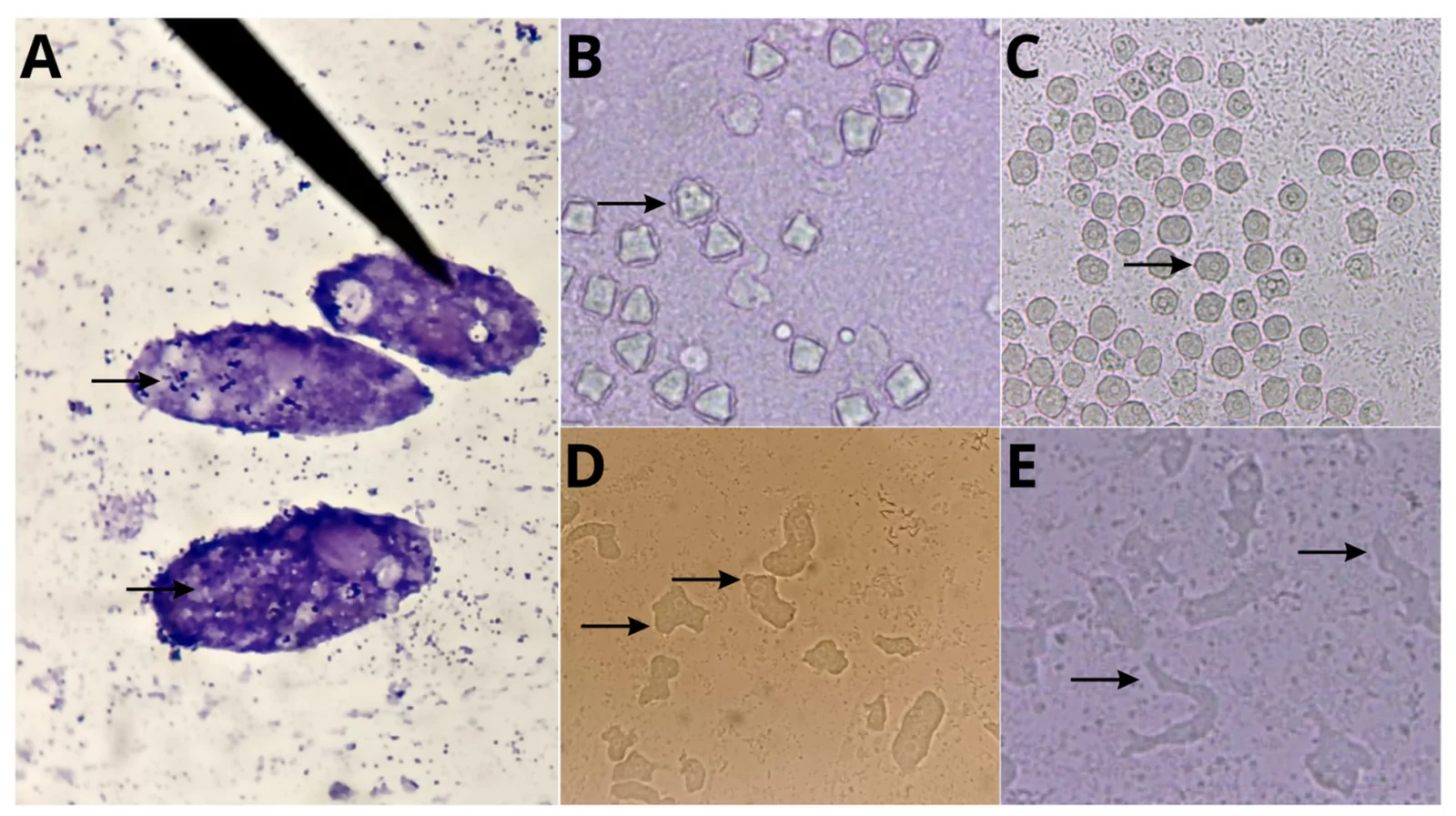
Morphological characteristics of cysts and trophozoites of free-living amoebae isolated from environmental water sources, with arrows indicating key diagnostic features. (**A**) *Acanthamoeba* spp.: Giemsa-stained trophozoites (40×) showing prominent contractile vacuoles (arrows). (**B**) *Acanthamoeba* spp.: mature cysts exhibiting the characteristic double wall with polygonal endocysts (arrow). (**C**) *Vermamoeba vermiformis*: ovoid and spherical single-walled cysts clustered in the culture medium (arrow). (**D**) *Acanthamoeba* spp.: live trophozoites in culture displaying characteristic acanthopodia (arrows). (**E**) *Vermamoeba vermiformis*: trophozoite exhibiting the characteristic elongated (limax-type) morphology (arrows).

**Figure 3:**
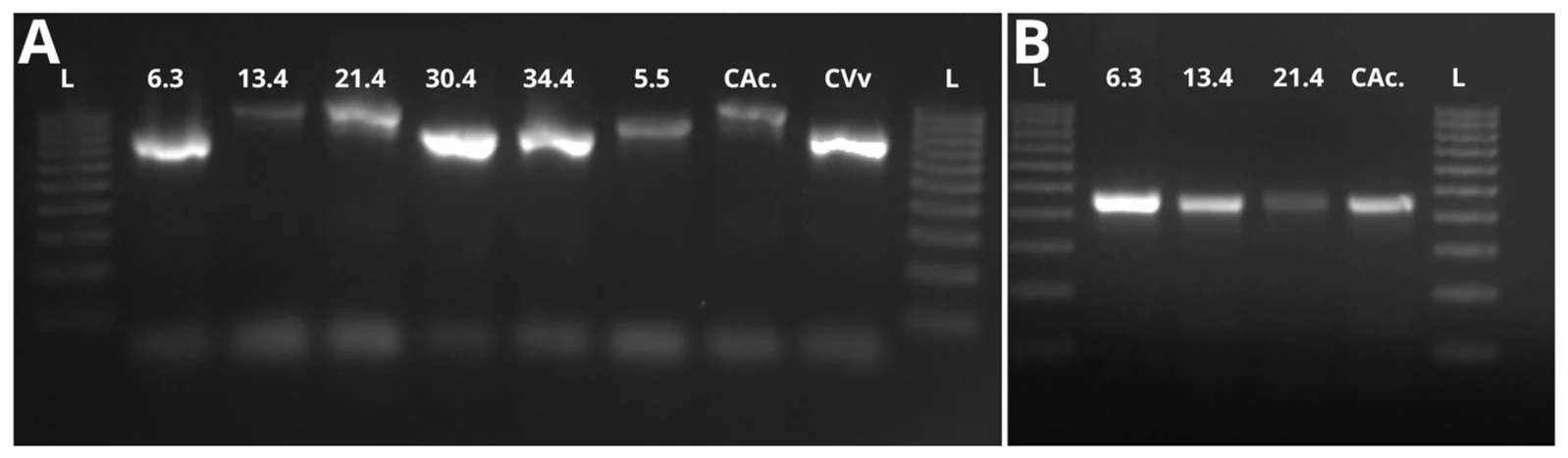
Molecular identification and characterization of free-living amoebae by PCR. (**A**) A 2% agarose gel showing amplification of a partial fragment of the 18S rRNA gene using the universal FLA primers. Differences in amplicon size are observed between *Acanthamoeba* spp. isolates (lanes 6.3, 13.4, and 21.4) and *Vermamoeba vermiformis* isolates (lanes 30.4, 34.4, and 5.5), alongside internal positive controls for each genus (CAc., CVv). (**B**) Amplification of the DF3 region of the 18S rRNA gene in *Acanthamoeba* spp. isolates (lanes 6.3, 13.4, and 21.4) using the JDP1/JDP2 primer set, alongside the internal positive control (CAc.). CAc.: internal positive control for *Acanthamoeba* spp. (isolate 3.2). CVv: internal positive control for *Vermamoeba vermiformis* (isolate 37.4). L: 100-bp DNA ladder.

**Figure 4:**
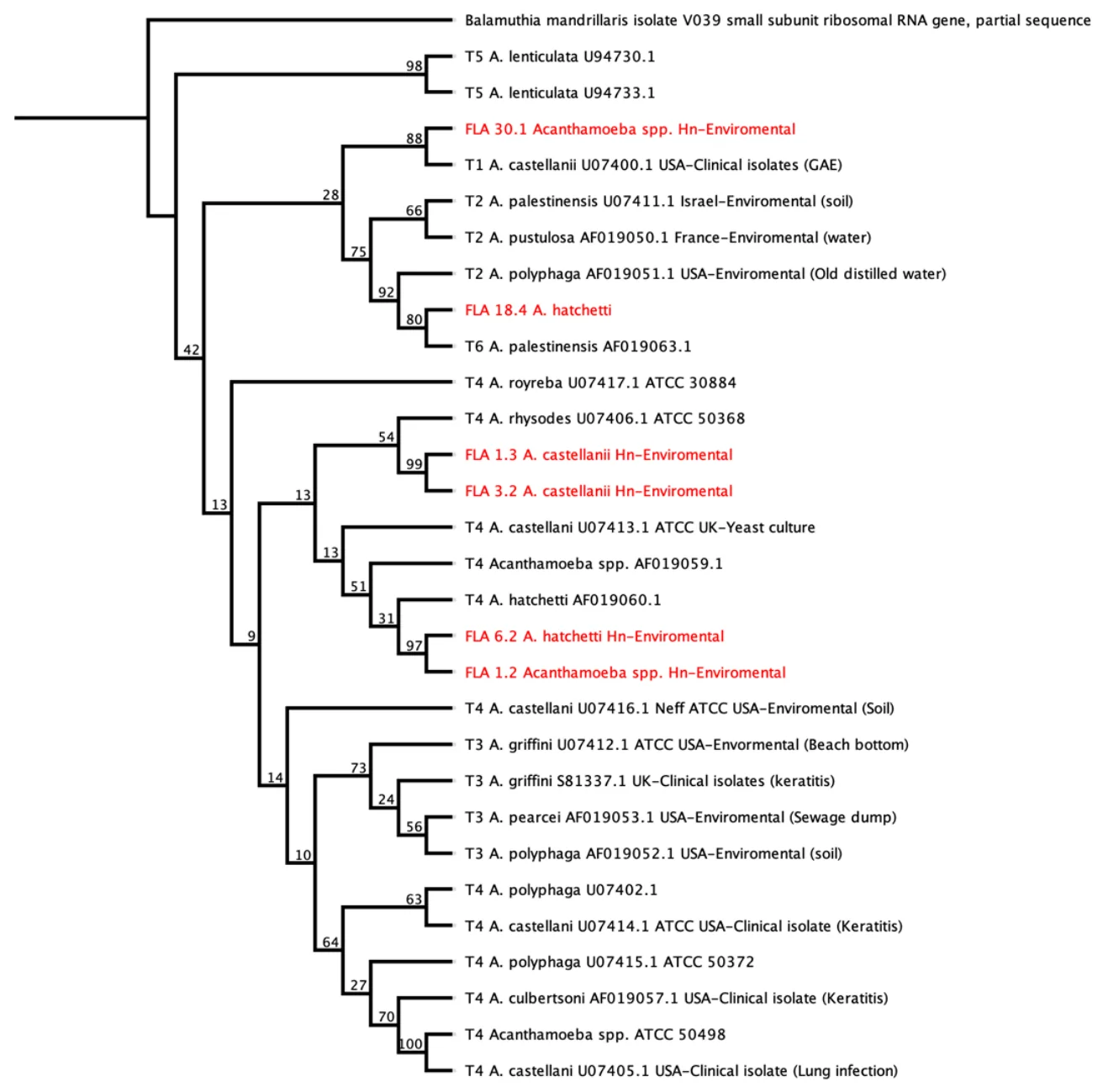
Maximum Likelihood phylogenetic tree (RAxML, GTR+GAMMA model, 1000 rapid bootstrap replicates) based on partial 18S rRNA gene sequences of *Acanthamoeba* spp. isolates obtained using the FLA-F/FLA-R primer set (highlighted in red). The tree was rooted with *Balamuthia mandrillaris* isolate V039 (GenBank accession number AF477019) as the outgroup. Numbers at the nodes indicate bootstrap support values; only values ≥ 50% are shown.

**Figure 5:**
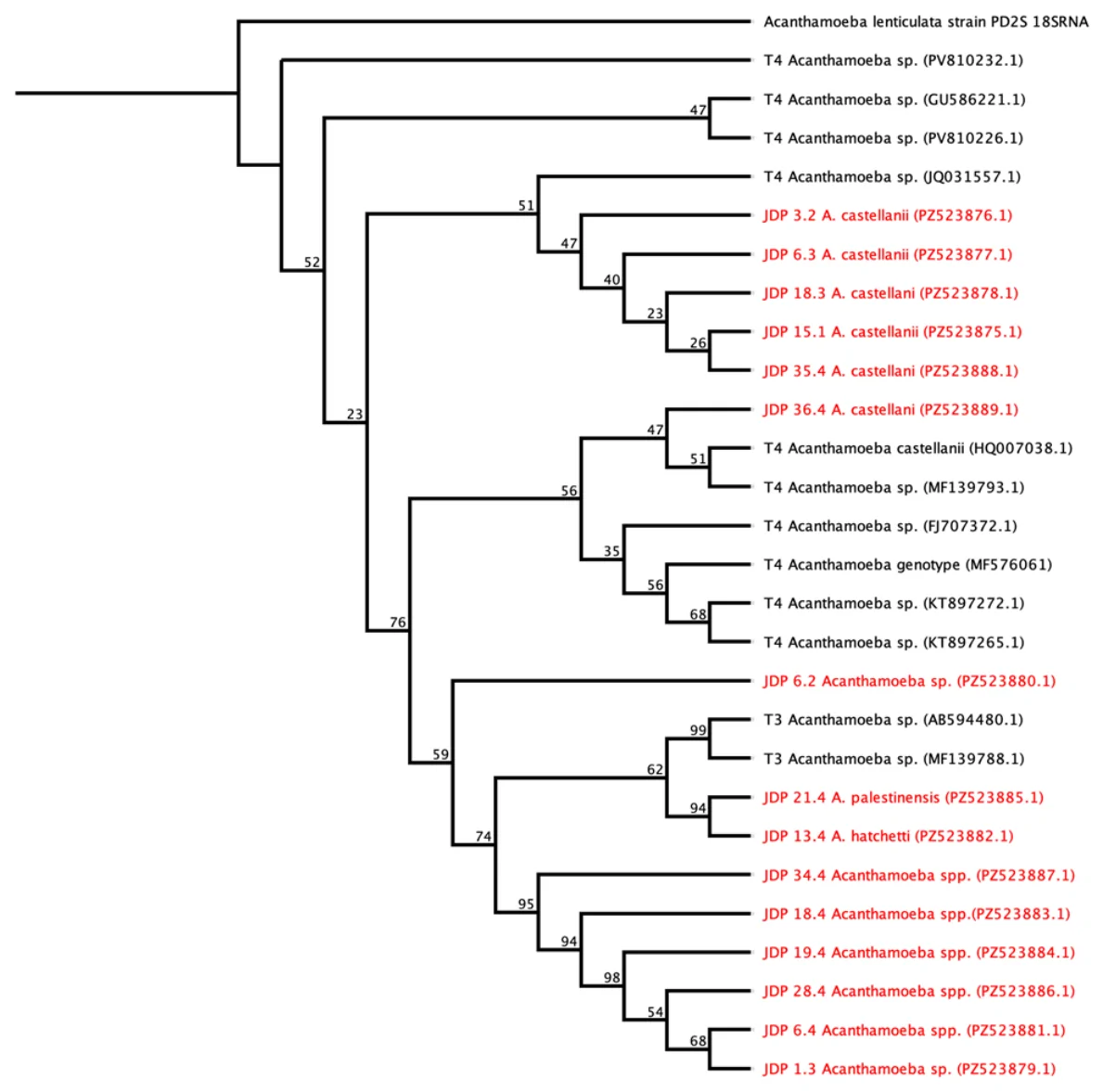
Maximum Likelihood phylogenetic tree (RAxML, GTR+GAMMA model, 1000 rapid bootstrap replicates) based on the hypervariable DF3 region of *Acanthamoeba* spp. isolates amplified with the JDP1/JDP2 primer set (highlighted in red). Because this short, genus-specific fragment could not be reliably aligned against a distantly related outgroup, the tree was rooted using *Acanthamoeba lenticulata* strain PD2S (genotype T5; GenBank accession number U94741), identified as the most basal genotype in Figure 4. Numbers at the nodes indicate bootstrap support values; only values ≥ 50% are shown.

**Figure 6:**
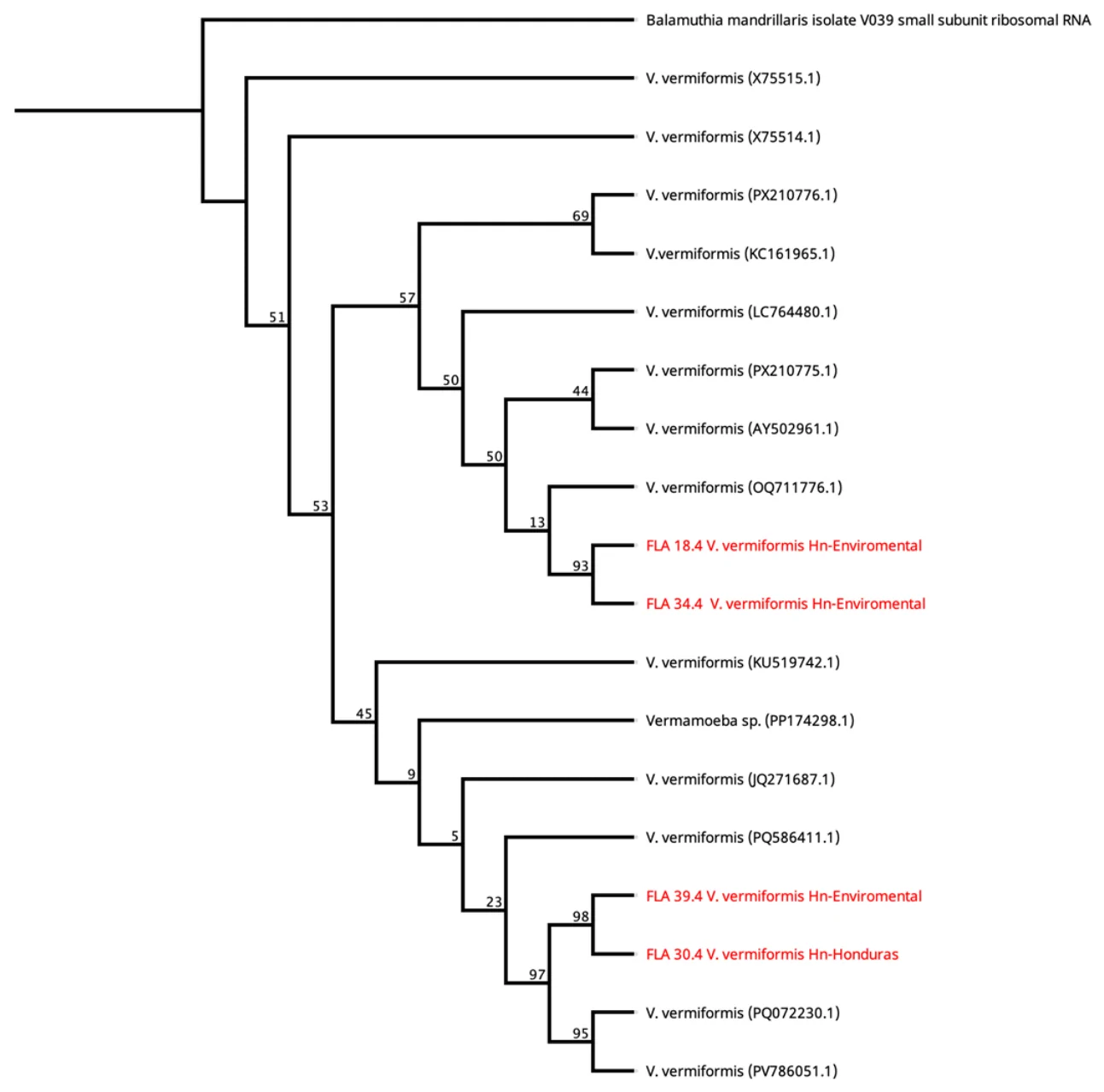
Maximum Likelihood phylogenetic tree (RAxML, GTR+GAMMA model, 1000 rapid bootstrap replicates) based on partial 18S rRNA gene sequences of *Vermamoeba vermiformis* isolates obtained using the FLA-F/FLA-R primer set (highlighted in red), rooted with *Balamuthia mandrillaris* isolate V039 (GenBank accession number AF477019) as the outgroup. Numbers at the nodes indicate bootstrap support values; only values ≥ 50% are shown.

**Table 1:** Primers used for the molecular identification of free-living amoebae (FLA).

Target Organism	Primers	Sequence (5′–3′)	*T_m_* (°C)	Reference
Free-living amoebae (universal)	FLA-F	CGCGGTAATTCCAGCTCCAATAGC	50	[21]
	FLA-R	CAGGTTAAGGTCTCGTTCGTTAAC		
*Acanthamoeba* spp.	JDP1	GGCCCAGATCGTTTACCGTGAA	50	[22]
	JDP2	TCTCACAAGCTGCTAGGGAGTCA		

**Table 2:** Molecular and physiological characterization of free-living amoebae isolated from environmental water sources in Honduras.

No.	Code	Sampling Site	Coordinates		Water Source	Taxonomic Identification	Thermotolerance			Osmotolerance		GenBank Accession Number
			Latitude	Longitude			37 °C (h)	42 °C (h)	45 °C (h)	0.5 M	1 M	
1	15.1	El Paraíso	14.177916	−86.880042	Tap water	*Acanthamoeba castellanii*	96	96	48	96	96	PZ523875
2	25.1	El Paraíso	14.183170	−86.883558	Tap water	*Acanthamoeba* sp.	48	-	-	96	96	
3	30.1	El Paraíso	14.181574	−86.877194	Stored water	*Acanthamoeba* sp.	96	-	-	96	72	PX462019
4	1.2	FM	14.049458	−87.099914	Stored water	*Acanthamoeba* sp.	96	-	-	96	96	PX462017
5	3.2	FM	14.049294	−87.100187	Stored water	*Acanthamoeba castellanii*	96	48	-	96	96	PX462020
6	6.2	FM	14.050166	−87.099426	River	*Acanthamoeba hatchetti*	72	-	-	96	72	PX462021
7	1.3	Valle	13.614151	−87.645469	River	*Acanthamoeba castellanii*	96	-	-	96	96	PX462018
8	6.3	Valle	13.618428	−87.655853	Tap water	*Acanthamoeba castellanii*	72	-	-	96	96	PZ523877
9	18.3	FM	14.085691	−87.166261	Tap water	*Acanthamoeba castellanii*	96	72	-	96	96	PZ523878
10	1.4	FM	14.084664	−87.165691	Tap water	*Acanthamoeba* sp. y *Vermamoeba vermiformis*	NA	NA	NA	NA	NA	
11	2.4	FM	14.084547	−87.165721	Tap water	*Acanthamoeba* sp.	96	-	-	-	-	
12	3.4	FM	14.084872	−87.167545	Tap water	*Acanthamoeba* sp. y *Vermamoeba vermiformis*	NA	NA	NA	NA	NA	
13	4.4	FM	14.084505	−87.167604	Tap water	*Acanthamoeba* sp. y *Vermamoeba vermiformis*	NA	NA	NA	NA	NA	
14	6.4	FM	14.083951	−87.167639	Tap water	*Acanthamoeba* sp.	96	-	-	96	96	PZ523881
15	7.4	FM	14.083915	−87.167223	Tap water	*Acanthamoeba* sp.	-	-	-	-	-	
16	8.4	FM	14.083860	−87.167797	Tap water	*Acanthamoeba* sp. y *Vermamoeba vermiformis*	NA	NA	NA	NA	NA	
17	9.4	FM	14.085307	−87.163237	Tap water	*Acanthamoeba* sp.	48	-	-	-	-	
18	10.4	FM	14.085244	−87.162752	Tap water	*Acanthamoeba* sp.	96	72	-	72	-	
19	12.4	FM	14.085200	−87.163296	Tap water	*Acanthamoeba* sp.	-	-	-	-	-	
20	13.4	FM	14.085411	−87.162725	Tap water	*Acanthamoeba hatchetti*	72	-	-	96	96	PZ523882
21	14.4	FM	14.086612	−87.165568	Tap water	*Acanthamoeba* sp. y *Vermamoeba vermiformis*	NA	NA	NA	NA	NA	
22	15.4	FM	14.086449	−87.165400	Tap water	*Vermamoeba vermiformis*	96	-	-	-	-	
23	16.4	FM	14.086406	−87.165296	Tap water	*Acanthamoeba* sp.	96	-	-	-	-	
24	17.4	FM	14.088829	−87.170464	Tap water	*Acanthamoeba* sp.	96	96	96	-	-	
25	18.4	FM	14.085700	−87.165015	Tap water	*Vermamoeba vermiformis*	96	-	-	96	-	PX584562
						*Acanthamoeba hatchetti*	96	-	-	48	-	PX930082.1
26	19.4	FM	14.085845	−87.164725	Tap water	*Acanthamoeba* sp.	72	-	-	96	72	PZ523884
27	20.4	FM	14.085762	−87.164833	Tap water	*Vermamoeba vermiformis*	96	48	-	96	-	
28	21.4	FM	14.086099	−87.166543	Tap water	*Acanthamoeba palestinensis*	96	96	48	96	-	PZ523885
29	22.4	FM	14.085938	−87.166409	Tap water	*Acanthamoeba* sp. y *Vermamoeba vermiformis*	NA	NA	NA	NA	NA	
30	23.4	FM	14.085850	−87.166765	Tap water	*Acanthamoeba* sp. y *Vermamoeba vermiformis*	NA	NA	NA	NA	NA	
31	24.4	FM	14.085056	−87.164542	Tap water	*Acanthamoeba* sp.	96	-	-	-	-	
32	26.4	FM	14.084833	−87.163951	Tap water	*Acanthamoeba* sp. y *Vermamoeba vermiformis*	NA	NA	NA	NA	NA	
33	27.4	FM	14.085065	−87.164239	Tap water	*Acanthamoeba* sp. y *Vermamoeba vermiformis*	NA	NA	NA	NA	NA	
34	28.4	FM	14.085586	−87.164310	Tap water	*Acanthamoeba* sp.	96	-	-	96	96	PZ523886
35	30.4	FM	14.085299	−87.163672	Tap water	*Vermamoeba vermiformis*	96	-	-	-	-	PX584563
36	33.4	FM	14.085086	−87.162207	Tap water	*Acanthamoeba* sp. y *Vermamoeba vermiformis*	NA	NA	NA	NA	NA	
37	34.4	FM	14.089632	−87.161894	Tap water	*Vermamoeba vermiformis*	96	-	-	-	-	PX584561
						*Acanthamoeba* sp.	96	72	-	96	-	PZ523887
38	35.4	FM	14.084994	−87.162053	Tap water	*Acanthamoeba castellanii*	96	-	-	96	96	PZ523888
39	36.4	FM	14.085530	−87.162116	Tap water	*Acanthamoeba castellanii*	96	-	-	96	72	PZ523889
40	37.4	FM	14.085603	−87.162308	Tap water	*Vermamoeba vermiformis*	-	-	-		-	
41	38.4	FM	14.085603	−87.162308	Tap water	*Vermamoeba vermiformis*	96	-	-	72	-	
42	39.4	FM	14.085163	−87.162352	Tap water	*Vermamoeba vermiformis*	96	-	-	72	-	PZ526876
43	5.5	Comayagua	14.497131	−87.641362	Tap water	*Vermamoeba vermiformis*	-	-	-	-	-	

FM: Francisco Morazán. (-): no growth. NA: not assessed (mixed culture or unpurified isolate). Isolates 3.2, 6.2, 18.4 (*Acanthamoeba*) and 1.3 also sequenced with JDP1/JDP2 (accessions PZ523876/80/83/79).

## Data Availability

The datasets generated and analyzed during the current study are available from the corresponding author upon reasonable request.

## Supplementary Materials

The following supporting information can be downloaded at https://www.mdpi.com/article/10.3390/tropicalmed11100271/s1: File S1: Sequence alignment used to construct the phylogenetic tree shown in Figure 4. File S2: Sequence alignment used to construct the phylogenetic tree shown in Figure 5. File S3: Sequence alignment used to construct the phylogenetic tree shown in Figure 6.
